# Association of Sicca Syndrome with Proviral Load and Proinflammatory Cytokines in HTLV-1 Infection

**DOI:** 10.1155/2016/8402059

**Published:** 2016-01-19

**Authors:** Clara Mônica Lima, Silvane Santos, Adriana Dourado, Natália B. Carvalho, Valéria Bittencourt, Marcus Miranda Lessa, Isadora Siqueira, Edgar M. Carvalho

**Affiliations:** ^1^Postgraduate Program in Health Sciences, Federal University of Bahia School of Medicine, 40025-010 Salvador, BA, Brazil; ^2^Immunology Service, Professor Edgard Santos University Hospital, Federal University of Bahia, 40110-060 Salvador, BA, Brazil; ^3^Department of Otolaryngology, Federal University of Bahia, 40110-060 Salvador, BA, Brazil; ^4^National Institute of Science and Technology of Tropical Diseases (CNPq/MCT), 40110-060 Salvador, BA, Brazil; ^5^Department of Biological Sciences, State University of Feira de Santana (UEFS), 44036-900 Feira de Santana, BA, Brazil; ^6^Gonçalo Moniz Research Center, Oswaldo Cruz Foundation (FIOCRUZ), 40296-710 Salvador, BA, Brazil

## Abstract

The* Sjögren* syndrome has been diagnosed in patients with HTLV-1 associated myelopathy and dry mouth and dry eyes are documented in HTLV-1 carriers. However the diagnosis of* Sjögren* syndrome in these subjects has been contested. In this cross-sectional study, we evaluated the role of immunological factors and proviral load, in sicca syndrome associated with HTLV-1 in patients without myelopathy. Subjects were recruited in the HTLV-1 Clinic, from 2009 to 2011. The proviral load and cytokine levels (IFN-*γ*, TNF-*α*, IL-5, and IL-10) were obtained from a database containing the values presented by the subjects at admission in the clinic. Of the 272 participants, 59 (21.7%) had sicca syndrome and in all of them anti-*Sjögren* syndrome related antigen A (SSA) and antigen B (SSB) were negatives. The production of TNF-*α* and IFN-*γ* was higher in the group with sicca syndrome (*P* < 0.05) than in HTLV-1 infected subjects without sicca syndrome. Our data indicates that patients with sicca syndrome associated with HTLV-1 do not have* Sjögren* syndrome. However the increased production of TNF-*α* and IFN-*γ* in this group of patients may contribute to the pathogenesis of sicca syndrome associated with HTLV-1.

## 1. Introduction

The human T lymphotropic virus type 1 (HTLV-1) infection is distributed worldwide with high prevalence in Central Africa, Central and South America, and South west of Japan [1]. Adult T cell leukemia/lymphoma (ATL), HTLV-1 associated myelopathy or tropical spastic paraparesis (HAM/TSP), and infective dermatitis are etiologically associated with HTLV-1 [2]. The HTLV-1 infects predominantly not only T cells but also B cells and myeloid cell lineage inducing cell activation and proliferation [3]. In such case it is possible that HTLV-1 infecting or activating autoreactive cells might induce the appearance of autoimmune diseases. Actually many reports in the last 20 years showed an association between rheumatoid arthritis, polymyositis,* Sjögren* syndrome, and systemic lupus erythematosus with HTLV-1 [4–6]. HTLV-1 infection was documented in up to 30% of patients with rheumatoid arthritis in endemic areas for this virus, and* Sjögren* syndrome was reported between 30 and 60% in patients who had HAM/TSP [7–9].* Sjögren* syndrome is a chronic autoimmune disease of the exocrine glands that affects the salivary and lacrimal glands through a lymphocytic infiltrate, leading to xerostomia (dry mouth) and xeroftalmia (dry eye). However, more recently the association between autoimmune diseases and HTLV-1 has been contested [10, 11]. There is no doubt that a large number of HTLV-1 infected individuals have dry mouth, dry eyes, and arthropathy, but synovitis and polyarthritis are rare [12, 13]. Lymphocyte infiltration has been documented in salivary glands of HTLV-1 infected subjects indicating its participation in the pathogenesis of salivary glands destruction [14]. But occurrence of autoantibodies characteristic of* Sjögren* syndrome was not observed in patients with the sicca syndrome associated with HTLV-1 infection [10]. Moreover arthritis is not a common finding in patients with sicca syndrome associated with HTLV-1 [15]. Therefore the pathogenesis of sicca syndrome related to HTLV-1 is not clear and the possibility that an autoimmune disease may account for the occurrence of it has been argued.

The role of the inflammatory response and proviral load in the pathogenesis of clinical manifestations related to HTLV-1 has been well documented. Proinflammatory cytokines and chemokines are higher in supernatants of peripheral blood mononuclear cells (PBMCs) culture and in serum of HAM/TSP than HTLV-1 carriers [16, 17] and there is an association of high proviral load with HAM/TSP [18–20]. In patients with HTLV-1 associated periodontal disease, mRNA for tax was present in the periodontal tissue and there was an increased expression of IL-1*β* and IFN-*γ* and a decrease in the expression of IL-10 and regulatory T cells in this tissue [21, 22]. Furthermore, both proviral load and production of proinflammatory cytokines are higher in patients with neurogenic bladder associated with HTLV-1 but who do not fulfill the criteria for HAM/TSP, as well as in children with infective dermatitis, than in HTLV-1 carriers, which indicates that these variables are associated with diseases related to HTLV-1 [23, 24]. The aim of this study was to evaluate if there was an association between the levels of cytokines, proviral load, and anti-*Sjögren* syndrome related antigen A (SSA) and anti-*Sjögren* syndrome related antigen B (SSB) antibodies with sicca syndrome associated with HTLV-1.

## 2. Material and Methods

### 2.1. Subjects and Diagnosis Criteria

This is a cross-sectional study comparing proviral load and cytokine levels among HTLV-1 infected subjects with or without sicca syndrome. Participants of this study include 272 HTLV-1 infected subjects with age range from 18 to 60 years, of both genders, followed at the HTLV-1 Multidisciplinary Clinic of the Hospital Universitário Professor Edgard Santos in Salvador, Bahia, Brazil. Subjects admitted to the clinic are referred from blood banks or from other clinics due to a positive serology for HTLV-1 and HTLV-2, by enzyme-limited immunosorbent assay (Murex HTLV-I + II Abbot, Dartford, UK). The diagnosis of HTLV-1 is confirmed by Western blot (HTLV Blot 2.4, Genelab, Singapore). The inclusion criteria for participation in the study were the presence of a positive serology for HTLV-1 confirmed by Western blot. Exclusion criteria were presence of human immune deficiency virus (HIV) and diagnosis of HAM/TSP based on the Osame motor disability score (OMDS) ≥ 1. Moreover 27 patients were excluded due to coinfection with hepatitis B or hepatitis C virus. All subjects answered a questionnaire regarding dry mouth and dry eyes and had clinical examination. Dry mouth was determined by oral examination and the salivary flux by the Saxon test [25]. Sicca syndrome was defined by the documentation of dry mouth and abnormal Saxon test. The majority of the patients also complained of dry eyes.

### 2.2. Evaluation of Autoantibodies

Serum samples were screened for antinuclear antibodies by immunofluorescence and anti-SSA and anti-SSB by ELISA as previously described [26, 27].

### 2.3. Immunologic Studies

Cytokines were determined in supernatants of unstimulated PBMCs cultures as previously described [23]. Briefly, PBMCs were obtained from heparinized venous blood by density gradient centrifugation with Ficoll-hypaque (GE Healthcare Bio-Sciences Uppsala Sweden). The mononuclear cells were then washed in saline and after being adjusted to the concentration of 3 × 10^6^ cells/mL were resuspended in RPMI 1640 (Life Technologies Gibco BRL, Gran Island, New York) supplemented with 10% of fetal bovine serum and antibiotics. Unstimulated cells were incubated for 72 hours at 37°C 5% CO_2_ and the supernatants were harvested. Determination of IFN-*γ*, TNF-*α*, IL-5, and IL-10 was performed by ELISA using reagents from BD Biosciences Pharmingen, San Jose, CA.

### 2.4. HTLV-1 Proviral Load

DNA was extracted from 10^6^ PBMCs using proteinase K and salting-out method. The HTLV-1 proviral load was quantified using a real-time TaqMan PCR method as previously described using the ABI Prism 7700 Sequence detector system (Applied Biosystems) [28]. Albumin DNA was used as an endogenous reference. The normalized value of the HTLV-1 proviral load was calculated as the ratio of (HTLV-1 DNA average copy number/albumin DNA average copy number) × 2 × 10^6^ and expressed as the number of HTLV-1 copies per 10^6^ PBMCs.

### 2.5. Statistical Analysis

The comparison between the ages in the 2 groups was performed by Student's *t*-test. The comparison between proportions was performed by Fisher exact test. The data on cytokine levels and proviral load were expressed as median and interquartile (IQ) range and were analyzed by the Kruskal-Wallis test. The correlation between proviral load and cytokine levels was performed by the correlation of Spearmen. The GraphPad Prism 5 (San Diego, CA) was used to perform the statistical evaluation and *P* values < 0.05 were considered statistically significant.

## 3. Results

Of the 272 participants of the study, 59 (21.7%) had sicca syndrome. The age, gender, and ethnic group of HTLV-1 infected subjects with sicca syndrome and without sicca syndrome are shown in Table 1. There was no difference regarding age in the two groups (*P* = 0.847) and the female gender predominates in both groups without statistical significance. There were more blacks in the group with sicca syndrome (*P* = 0.03).

The diagnosis of sicca syndrome was based on oral examination and a reduction in the salivary fluid by Saxon test [25]. Regarding other diseases related to HTLV-1, overactive bladder a manifestation considered as an oligosymptomatic form of HAM/TSP was documented in 11 (18.6%) of the patients with sicca syndrome and in 26 (12.2%) in the patients without sicca syndrome (*P* > 0.5). There was also no difference regarding polyarthralgia in the 2 groups and synovitis was detected in only 2 patients. One patient had mixed connective tissue disease and sicca syndrome and the other had a seronegative rheumatic arthritis and did not have sicca syndrome.

**Figure 1:**
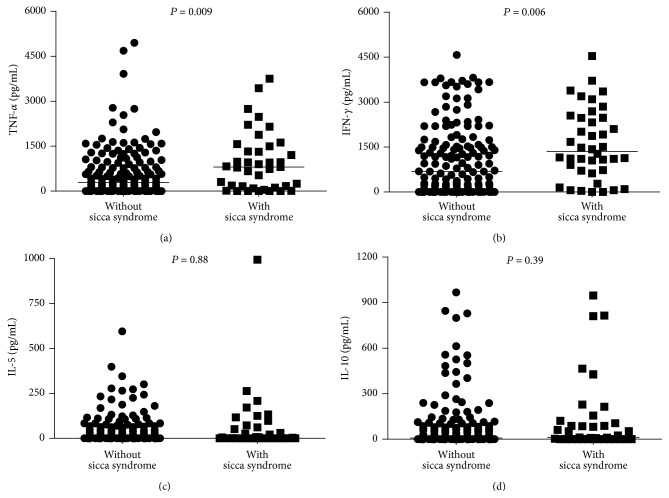
Levels of TNF-*α* (a), IFN-*γ* (b), IL-5 (c), and IL-10 (d) produced by PBMC from HTLV-1 infected individuals with and without sicca syndrome. Cytokines data represent the values in unstimulated cultures. The levels of cytokines were measured by ELISA.

The spontaneous cytokines (TNF-*α*, IFN-*γ*, IL-5, and IL-10) levels in supernatants of PBMCs are shown in Figure 1. The levels of TNF-*α* in patients with sicca syndrome (median 803 pg/mL, IQ range 116–1,498) were higher (*P* < 0.009, Figure 1(a)) than that observed in patients without sicca syndrome (median 281 pg/mL, IQ range 0–946). The production of IFN-*γ* in patients with sicca syndrome (median 1,352 pg/mL, IQ range 717–2,477) was higher (*P* = 0.006, Figure 1(b)) than in the group without sicca syndrome (median 682 pg/mL, IQ range 42–1,604). No difference was observed in the median of the IL-5 levels (*P* = 0.88, Figure 1(c)) in the group with sicca syndrome (1 pg/mL, IQ range 0–61) and that without sicca syndrome (median 0 pg/mL, IQ range 0–62). The production of IL-10 (Figure 1(d)) did not differ between groups (*P* = 0.39). Cytokine levels were undetectable or were very low in supernatants of PBMCs of patients with sicca syndrome.

**Figure 2:**
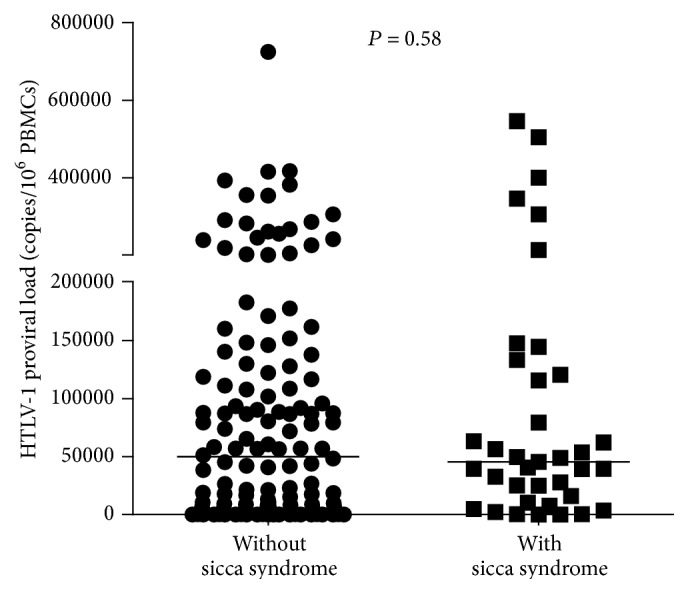
HTLV-1 proviral load of HTLV-1 infected individuals with and without sicca syndrome. Proviral load was quantified by real-time TaqMan PCR method and the normalized value of the proviral load was calculated as the ratio of (HTLV-1 DNA average copy number/albumin DNA average copy number) × 2 × 10^6^ and expressed as the number of HTLV-1 copies per 10^6^ PBMCs.

**Figure 3:**
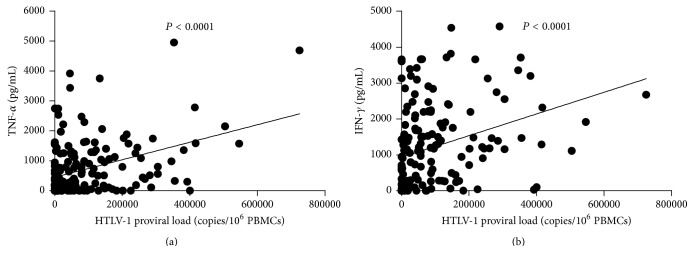
Correlation between proviral load and cytokine levels in HTLV-1 infected subjects. There was a direct correlation between proviral load and TNF-*α* levels (a) and between proviral load and IFN-*γ* levels (b) when data from all participants of the study were analyzed.

The proviral load in HTLV-1 infected subjects with and without sicca syndrome is shown in Figure 2. There was no difference between proviral load (*P* = 0.58) in patients with sicca syndrome (median 45,554, IQ range 13,171–126,803 copies/10^6^ cells) and in patients without sicca syndrome (median 49,861 copies/10^6^ cells, IQ range 2,184–128,187). There was a direct correlation between proviral load and IFN-*γ* and proviral load and TNF-*α* when values obtained in the whole sample were analyzed (Figure 3). However no correlation was observed when data from patients with sicca syndrome or without sicca syndrome were analyzed isolatedly.

Antinuclear antibodies and anti-SSA and anti-SSB antibodies were determined in the two groups. Anti-SSA and anti-SSB antibodies were absent in all subjects. Antinuclear antibodies were detected in 2 subjects, one in the group with and another in the group without sicca syndrome.

## 4. Discussion

In the present study we show that the proinflammatory cytokines IFN-*γ* and TNF-*α* were higher in HTLV-1 infected patients with sicca syndrome than in HTLV-1 infected subjects without sicca syndrome, indicating that the exacerbated proinflammatory response observed in HTLV-1 infection may play a role in the destruction of the salivary and lacrimal glands observed during this viral infection. Moreover our data indicate that autoimmune rheumatic diseases are rarely associated with HTLV-1 and that there is no evidence of* Sjögren* syndrome in HTLV-1 infected subjects without HAM/TSP.

The prevalence of* Sjögren*-like syndrome in HTLV-1 infected subjects ranges in accordance with the population studied. Initial studies showed that this association was mainly found in patients with HAM/TSP [7, 8]. However in a cross-sectional study evaluating the frequency of clinical manifestations in HTLV-1 carriers and in seronegative controls, the prevalence of dry mouth was 20.8% in carriers, while it was 11.3% in non-HTLV-1-infected subjects [13]. Poetker et al. also showed a prevalence of 22.5% of dry mouth in HTLV-1 carriers referred from blood banks recently diagnosed with HTLV-1 [12]. Herein, based on the complaint and documentation of dry mouth in the oral examination and a decrease in salivary output determined by Saxon test, the frequency of sicca syndrome in HTLV-1 infected subjects without HAM/TSP was similar to that previously found in a study performed in a HTLV-1 Clinic with small number of participants [29]. HAM/TSP and ATL are the more severe diseases related to HTLV-1, but other recognized manifestations associated with this viral infection include uveitis, chronic periodontitis, urinary manifestations of overactive bladder, sicca syndrome, and HTLV-1 associated arthropathy [2, 13, 30, 31]. It is worthwhile to emphasize that for many years HTLV-1 infection was considered a low morbidity infection as less than 5% of the infected subjects develop HAM/TSP or ATL. In this study we showed that sicca syndrome is frequent even in patients without HAM/TSP.

The documentation of a lymphocytic infiltration and the tax gene expression in the salivary gland of patients with dry mouth infected by the virus are the main evidences that salivary gland destruction in HTLV-1 infection is mediated by T cells [21, 32, 33]. Additionally HTLV-1 was expressed in salivary gland of transgenic mice that express the tax gene and presents a picture similar to* Sjögren* syndrome [34]. However more recently as autoantibodies related to* Sjögren* syndrome have not been documented in such patients, the occurrence of* Sjögren* syndrome associated with HTLV-1 has been argued [10, 35]. Herein patients with dry mouth and dry eyes did not present either anti-SSA or anti-SSB antibodies. This gives support to the concept that, rather than* Sjögren* syndrome, HTLV-1 infected subjects have a sicca syndrome due to destruction of the salivary glands.

The pathogenesis of the clinical manifestations related to HTLV-1 has been mainly studied in patients with HAM/TSP and is likely multifactorial. The neurologic disease has been associated with high proviral load [18–20] and increased levels of proinflammatory cytokines including IL-1, IL-6, TNF-*α*, and IFN-*γ* [36, 37]. In such case there is a passage of T cells from blood to the central nervous system and the tissue damage is mediated by an exaggerated and nonmodulated immunologic response [38, 39]. Giving support to the role of an exaggerated inflammatory response in the pathogenesis of manifestations due to HTLV-1, high levels of proinflammatory cytokines have been documented in other diseases associated with HTLV-1, as in patients with neurogenic bladder who do not fulfill the criteria for HAM/TSP [23], in patients with chronic periodontal disease associated with HTLV-1 [22], and in children with infective dermatitis, a disease associated with development of HAM/TSP [24].

The previous documentation that salivary glands in HTLV-1 infected subjects are infiltrated by lymphocytes and the present study showing an increased production of TNF-*α* and IFN-*γ* in patients with HTLV-1 associated with sicca syndrome indicate that viral factors and an increased inflammatory response participate in the pathogenesis of sicca syndrome associated with HTLV-1.

## Figures and Tables

**Table 1:** Demographic characteristics of patients with and without sicca syndrome associated with HTLV-1 infection.

	Without sicca syndrome		With sicca syndrome		*P* value
	(*n* = 213; 88.3%)		(*n* = 59; 21.7%)		
Gender					0.100
Male, *n* (%)	94	44.1%	19	32.2%	
Female, *n* (%)	119	55.9%	40	67.8%	
Age	Mean	SD	Mean	SD	0.847
	46.90	12.13	46.54	14.22	
Race					0.03
White, *n* (%)	48	23.2%	13	23.6%	
Mulate, *n* (%)	94	45.4%	15	27.3%	
Black, *n* (%)	62	30.0%	27	49.1%	
Other, *n* (%)	3	1.4%	0	0.0%	
